# My Leadership Engine

**DOI:** 10.3389/fpubh.2015.00137

**Published:** 2015-05-11

**Authors:** Marina Binet Baroff

**Affiliations:** ^1^Community Information Exchange San Diego, San Diego, CA, USA

**Keywords:** gender equity, lessons learned, leadership, women in leadership

As a senior healthcare executive and fellow in the American College of Healthcare Executives (ACHE), I am often asked by junior colleagues how I became a leader. Since many women administrators still encounter difficulty in breaking through the glass ceiling to the executive suite, gender equity and personal values are frequently central to my narrative. Although more recently characterized as a labyrinth, which is neither simple nor easy to navigate, the glass ceiling, a visible, but clear and impenetrable barrier continues to prevent women from executive advancement (1). Thus, for many female leaders, negotiating a path to an executive position requires persistence and self-awareness. In describing my own career trajectory, which culminated in a position as a Chief Operating Officer, I emphasize the useful lessons which shaped my leadership behavior and the learnings that can serve as helpful hints for early careerists.

Since my career path spanned work in a local health department, clinics, hospitals, medical groups, and health plans, I frequently address the mix of agencies by highlighting my dual interests in macro policy development and micro level operations. As a female senior executive, faced with countless gender expectations, I stress the critical importance of promoting women into leadership roles. Within the hospital industry where women comprise more than 75% of the workforce, yet men are 74% of the chief executives; the opportunities for women’s advancement to the c-suite remain limited (2).

Early in my work-life, though there was an occasional nun or former nurse who stood at the helm of a large healthcare organization, most women leaders were clustered in department head or senior leadership roles. The executive suite was not entirely restricted; rather, women were underrepresented in general management and tended to fill nursing, planning, and marketing roles, not traditional pathways to executive advancement (3). This double standard played out on a daily basis, when male colleagues were praised for bold, visionary thinking, while women were chided for aggressive outspokenness. As a result, when it came time for CEO recruitment, boards of directors and other governing bodies tended to hire people who looked and spoke like them, typically meaning senior white males. Promotional opportunities that did exist were relatively few, and often demanded frequent moves, long-distance commuting, or family re-location, which were difficult for two career couples.

Twenty five years later, despite the progress made in educating and promoting women, this longstanding imbalance endures. Recent research indicates that men advance to hospital CEO positions at twice the rate of their female counterparts (2). In addition, since women constitute only 17% of top US corporate boards of directors, interviewing with male dominated executive boards continues to challenge women today (4). This stark reality contrasts with the perception held by many that because discrimination based on gender is illegal, the issue of gender bias or gender equity no longer exists. In fact, some men even claim that “although things might have been bad in the past, everything is fine now” (5).

Three decades ago, in nearly every healthcare setting, executive women mentors were either unseen or unknown. Consequently, my female colleagues relied upon our male bosses or female peers for career advice. While this informal support was helpful, mutual aims of professional advancement and work-life balance remained complex and unresolved.

By choice, necessity, and a commitment to lifelong learning, I pursued self-reflection as a means to better understand the challenges, situations, and opportunities I encountered as a woman healthcare professional. To assist leaders at all levels of responsibility, and to examine their philosophy, values, and behavior, *The Leadership Engine*, a book by Noel Tichy, Professor at the University of Michigan School of Business and former head of General Electric leadership training, provides a useful tool called, “the Teachable Point of View” (6).

Deceptively simple, this four step tool charts key nodal or life events on a timeline with positive, affirming experiences shown above the line, and challenges, failures, and unhappy incidents recorded below. The second step involves reflecting on these events relative to both favorable and disappointing outcomes and searches for recurring themes and repeated circumstances. Step three considers the principles and lessons learned and how these events influence leadership. The final step demands careful reflection and candor, sharing the self-assessment with others.

In applying this approach, six themes emerged in my timeline including: Projection, Preparation, Perseverance, Parity, Proof, and Play. Together, these six Ps constitute the fundamentals that fuel my leadership engine and serve as lessons learned that ultimately guided my leadership behavior. As such, they may provide a helpful roadmap that can be emulated by others.

## Projection

The first theme that resonated throughout crucial points in my life, correlated with my improbable career aspirations in espionage, which stemmed from my study of Russian history and language, and my fondness for reading mysteries. Projecting myself into the plotline in an effort to solve the problem presented, I also served as the “resident office futurist,” developing long-range plans, and forecasting business threats and opportunities. Addressing tough leadership questions with honesty and authenticity, and understanding the needs and concerns of others with a commitment to investigate and follow-up were also important precepts. Projection also called for creating an aura of poise and calm in the place of nervousness that might arise in addressing a large audience of professionals.

Futurist that I am, as a leader, I still face the never-ending trial of paying attention to what is in front of me, “staying in the moment.” Though much of my projection centered on strategic analysis and contingency planning, my propensity to look ahead may have obscured choices that were right before me and resulted in a missed learning opportunity. In the end, most decisions are a mixed blessing carrying both advantages and disadvantages.

## Preparation

The second theme powering my nodal event timeline is *preparation*. This need for readiness and implicit desire for control is, in part, a reaction to the sex-role stereotyping I encountered as a young girl. At that time, I learned that to succeed as a female in school, I had to work harder.

While performing as a top student and high achiever was not a struggle, it did require daily diligence and effort. Driving me to complete work in advance, practice everything, and rehearse repeatedly, I sometimes arrived in the classroom, office, or event over-prepared. Ironically, this careful planning often set the standard and modeled the way for others to follow. Given the inevitability of unforeseen mishaps, this groundwork allowed for flexibility and the freedom to improvise when necessary. Whether at work or play, drafting a presentation, rehearsing a speech, or running a marathon, formulating a game-plan in advance, and executing it in a step-wise fashion remains a core value and another lesson learned.

## Perseverance

A manifestation of my “stick-to-it-iveness,” and the third element in my leadership engine, means that I survey the long road ahead, forge a plan, and progress forward; even on the occasion when this doggedness proves a detriment. Since every strength, when taken to an extreme, can become a weakness, learning to temper my tenacity earlier in my career would have been helpful. In retrospect, there were times when taking a less far-reaching position might have been a more productive stance; particularly, when the progress of team goals was impeded by my persistence or reluctance to yield.

Due to a traumatic life event, which occurred in my mid-twenties, my tendency toward perseverance was really deep-seated in fear. From this pivotal experience, I adopted a new mantra of “feel the fear and do it anyway.” Ultimately, this incident with its polar opposite emotional amalgam of fear and courage led to a westerly migration for graduate school, which was not only liberating, but also served as the anchor for my subsequent family life and healthcare administration career.

## Parity

Synonymous with equity and fairness, *parity*, component number five, functioned as the primary catalyst for my active participation in the women’s movement as a feminist and believer in equal opportunity. Growing up in the South, in a Jewish home with an appreciation for the importance of justice and mercy, at a young age, I became aware of racial, religious, and gender discrimination. Observing its expression in my youth and college years, I developed a thick skin and fighting attitude toward inequality. I warmly recall protesting in national marches on behalf of women’s rights, and donning a suffragette ensemble to demonstrate at one state legislature for the Equal Rights Amendment. Witnessing injustice not only reaffirmed my advocacy for the underdog, but also strengthened my determination to treat all individuals as equals and view subordinates, peers, and managers as customers deserving of courtesy and mutual respect.

## Proof

Another predominant feature of my teachable point of view represented achievement, driving for results, and winning of recognition. Integral to the development of self-esteem, this need balanced the hunger for pride with the call for humility. While my competitive nature to prove my worth was reinforced at home and at school, if unchecked, it could create friction in the workplace by appearing too self-serving.

Despite the satisfaction I experienced from knowing that I was smart, I had enough self-awareness to recognize that I was less than perfect. Nevertheless, outside the office, I did excel as a member of the ACHE national Board of Governors. In this role, I visited more than eight states and met with other healthcare executives for mentoring, public speaking, and continuing education. Prior to advancing to the Board, I served as an elected leader for a women’s healthcare administration network and helped merge this organization with a local ACHE affiliate. When the local ACHE Regent unexpectedly moved out of the area, I was asked to step in as Interim Regent. This progression was followed in short order by election to Regent and an appointment as Governor for the Western Region of the United States. After my 4-year term on the Board concluded, I also served on the National Nominating Committee helping to choose the slate of future officers, and on the Chapters Committee which created the ACHE unified membership structure.

## Play

The last element of my six Ps equated to never surrendering my leisure time. Though I disciplined myself early on to finish assignments, I always made time for fun. Whether reading, walking, shopping, running, antiquing, writing, or watching old movies, reserving time to play was a priority. Even at the office where critical decisions were debated for hours, I injected lightheartedness into serious tasks. Once I choreographed a live auction using play Monopoly money while department managers bid on gift baskets for prizes tied to budget targets.

As I contemplate my own leadership engine and the lessons learned from my six Ps, I am reminded of both successes and disappointments as the price we pay for seeking challenging goals. On the one hand, though receiving frequent acknowledgments as an outstanding visionary leader, when I sought career advancement, internal promotional policies requiring geographic re-location frustrated my multiple attempts to break through the shatterproof, shock-resistant, Plexi-glass ceiling. Only after leaving a job of 20-plus years in one integrated health system and joining another, did I advance to the executive suite. Despite my hard work to lead a financial turnaround in a failing business unit and pilot an innovative project that achieved national recognition, I was laid off twice in two separate administrative restructures. Now, I am applying my leadership skills to building bridges across agencies and improving operations in the non-profit arena.

Thus, with my six Ps inter-weaving future planning, doing homework, sticking with it, promoting fairness, striving for results, and taking time out for renewal, my leadership journey continues. As I move forward on my own path, I will also continue to advocate for the advancement of women leaders. With gender equity in executive leadership, a business and moral imperative, all senior leaders must understand the issue and act to mitigate the inclination to bias and unfairness. To further collective progress, executive women at all levels of authority must take the initiative to mentor others. Employers need to embed professional development training, flexible work schedules, well-defined advancement criteria, and formal succession planning into the fabric of their organizational cultures. Educators should add personal values and professionalism into academic training, and create course offerings that assist all leaders to be more effective speakers and advocates on committees and workgroups. Ultimately, governing boards must display the courage to hire executives that look, think, and behave differently from the comfort-zone archetype of “male, pale, and stale” (7).

Checking in at a mid-west hotel, a desk clerk once glanced at my ACHE Governors badge, and asked if I was a Democrat or Republican? Struck speechless, but always ready for a bit of fun, I joked that I was an Independent from the State of Grace! So intending humor, maybe on that day, between being a former COO and a State or ACHE “Governor,” I had finally reached the pinnacle of my career. Perchance that glass ceiling was not so unbreakable after all!

I highly recommend the use of *The Leadership Engine* for all aspiring young executives, both men and women alike. Utilizing the four steps of “The Teachable Point of View,” charting nodal life events and reflecting on lessons learned can offer early careerists, an opportunity to assess current status and map a clearer pathway to success.

## Conflict of Interest Statement

The author declares that the research was conducted in the absence of any commercial or financial relationships that could be construed as a potential conflict of interest.
